# Cellular Mechanisms in Acute and Chronic Wounds after PDT Therapy: An Update

**DOI:** 10.3390/biomedicines10071624

**Published:** 2022-07-07

**Authors:** Vieri Grandi, Alessandro Corsi, Nicola Pimpinelli, Stefano Bacci

**Affiliations:** 1 Department of Health Sciences, Division of Dermatology, University of Florence, 50100 Florence, Italy; vieri.grandi@unifi.it (V.G.); pimpi@unifi.it (N.P.); 2 Guy’s and ST Thomas’NHS Foundation Trust, St John’s Institute of Dermatology, London SE17EP, UK; 3 Simple Unit of Vulnology, S. Raffaele Hospital, 20100 Milan, Italy; corsi.alessandro@hsr.it; 4 Research Unit of Histology and Embriology, Department of Biology, University of Florence, 50100 Florence, Italy

**Keywords:** 5-aminolevulinic acid (ALA), angiogenesis, acute wounds, cellular infiltrate, chronic wounds, mast cells, photodynamic therapy, nerves, neurons, wound healing

## Abstract

PDT is a two-stage treatment that combines light energy with a photosensitizer designed to destroy cancerous and precancerous cells after light activation. Photosensitizers are activated by a specific wavelength of light energy, usually from a laser. The photosensitizer is nontoxic until it is activated by light. However, after light activation, the photosensitizer becomes toxic to the targeted tissue. Among sensitizers, the topical use of ALA, a natural precursor of protoporphyrin IX, a precursor of the heme group, and a powerful photosensitizing agent, represents a turning point for PDT in the dermatological field, as it easily absorbable by the skin. Wound healing requires a complex interaction and coordination of different cells and molecules. Any alteration in these highly coordinated events can lead to either delayed or excessive healing. The goal of this review is to elucidate the cellular mechanisms involved, upon treatment with ALA-PDT, in chronic wounds, which are often associated with social isolation and high costs in terms of care.

## 1. The Photodynamic Therapy

In 1903, Von Tappeiner, in collaboration with Jesionek, demonstrated the therapeutic action of light combined with a photosensitizer and oxygen, and coined the term “Photodynamic action” [1]. Since that time, many researchers have experimentally verified the veracity of the efficacy on different biological structures. In medicine, the use of PDT is now widely documented and well-codified for the treatment of oncological and non-oncological diseases. In dermatology, the use varies from oncological pathologies such as basal cell carcinoma, squamous cell carcinoma, actinic and non-oncologic keratoses, bacterial, fungal, viral, immunological or inflammatory infections, to the treatment of chronic wounds, and finally, cosmetology for photorejuvenation [2,3,4,5]. PDT is based on the cytotoxic action of some hyperactive oxygen species (i.e., a type of unstable oxygen molecule that easily reacts with other molecules in a cell; a build-up of reactive oxygen species in cells may cause damage to DNA, RNA, and proteins, and potentially induce cell death [6]), especially singlet oxygen, but also superoxide anions and hydroxyl radicals, generated by the transfer of energy and/or electrons from the photoexcited oxygen sensitizer. Three important mechanisms are responsible for the efficacy of PDT: (1) direct death, or inflammation, of tumor cells, (2) damage to tumor vessels, (3) immunological response associated with the stimulation of leukocytes and release of interleukins and other cytokines, growth factors, complement components, acute phase proteins, and other immunoregulators [2,3,4,5]. In wound healing, recent studies show the efficacy of PDT for its antibacterial activity, in attacking the biofilm, and in remodeling the extracellular matrix by activating MMPs, thus inducing changes in the collagen of the extracellular matrix for the tissue healing process. In addition, PDT induces cellular changes, which is the phenomenon observed during the course of tissue repair [2,3,4,5].

## 2. Photosensitizers

PDT is a treatment that uses a photosensitizer (administered topically or systemically), light (which interacts with the substance in question), and oxygen to cause selective cell death by necrosis or apoptosis of the cells “atypically” sensitized, in which the photosensitizer or its precursor—administered topically or intravenously—accumulate selectively.

In summary, the photodynamic effect (through photophysical, photochemical, and photobiological mechanisms) is mediated by the generation of ROS, a process that depends on the intracellular interactions of the photosensitizer with light and oxygen [2,3,4,5]. 

**Figure 1:**
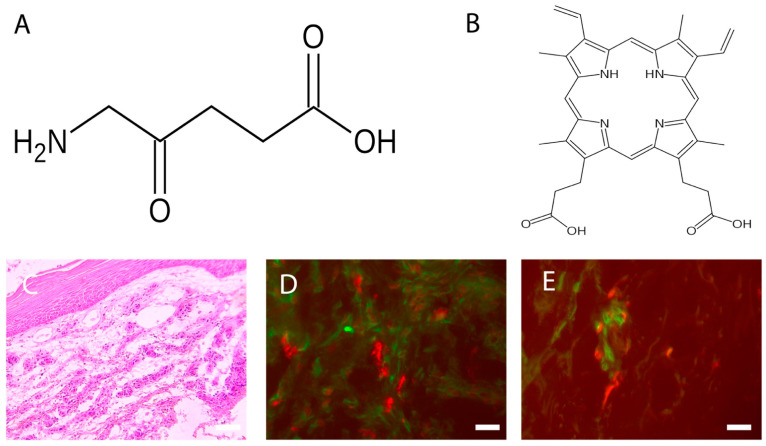
(**A**) Fvasconcellos (own work): Structural diagram of aminolevulinic acid. Created using ACD/ChemSketch 10.0 and Inkscape. This image of *a simple **structural formula*** is **ineligible for copyright** and, therefore, is in the **public domain**, because it contains no original authorship. (**B**) Fvasconcellos (own work): Skeletal formula of protoporphyrin IX. Created using ACD/ChemSketch 10.0 and Inkscape. The copyright holder of this work has released it into the public domain. This standard applies worldwide. In some countries this may not be legally possible. I grant anyone the right to use this work for any purpose, without any conditions, unless such conditions are required by law. (**C**) Chronic wound: Increased thickness of the epidermis and richness of cellular infiltrate. Hematoxylin Eosin, Light microscopy, scale bar = 10 microns. (**D**) Colocalization between MCs (stained with avidin, in red) and fibroblasts (stained with HSP47, in green) in PDT-treated chronic wounds. Fluorescence microscopy, scale bar =10 microns (see Table 1 for others information). (**E**) Colocalization between MCs (stained with avidin, in red) and DCs (stained with MHC class II, in green) in PDT-treated chronic wounds. Fluorescence microscopy, scale bar =10 microns (see Table 1 for others information).

The topical use of ALA (Figure 1A), a natural precursor of protoporphyrin IX (Figure 1B) and, in turn, a precursor of the heme group and a powerful photosensitizing agent, represents an important turning point in the dermatological field, as it is easily absorbable by the skin [4,5,7,8,9,10]. At the cellular level, the pro-drug, once transformed into protoporphyrin IX, causes the production of reactive oxygen species, which induce cell death in target cells. The presence of ROS in the immediate vicinity of cellular and subcellular membranes (in particular the mitochondrial ridges) allows the release of cytochrome C, with consequent activation of the caspase cascade, which ultimately leads to the intrinsic apoptotic phenomenon. The effect is enhanced by the degeneration of small vessels via a photodynamic mechanism, and by the triggering of an inflammatory reaction [5,6,7,8,9]. The concentration of 5-ALA usually depends on the mode of treatment, but the range is between 2–40% systematically, and 30–50 mg/cm2 topically. It is usually applied for less than 4 h, and it reaches peak accumulation between 3 and 8 h [4,5,7,8,9,10].

## 3. Wound Healing

Wound healing makes organisms resilient to injuries, allowing survival [14]. This process involves the interaction of various elements, such as soluble mediators (such as cytokines and factors growth), the extracellular matrix, vessels, and various other cell types. The physiological process underlying tissue repair is traditionally divided into four phases: coagulation, inflammatory, proliferative, and maturation [15,16,17,18,19,20,21,22]. 

***Coagulation phase:*** An initial process occurs during the inflammatory phase of hemostasis, with temporary vasoconstriction caused by release of vasoactive substances by damaged cells, followed by hemorrhage and subsequent platelet aggregation. The platelets, as well as being involved in clot formation, are also important producers of cytokines used in the activation of leukocytes and macrophages. With the aggregation process of the platelets, a biochemical cascade is then activated, in which dozens of factors are involved that lead to formation of an insoluble fibrin network [15,16,17,18,19,20,21,22]. 

***Inflammatory phase****:* The initial vasoconstriction process is followed by vasodilation mediated by substances such as histamine and serotonin secreted by MCs [23,24,25]. This leads to increased blood flow in the area of the wound that determines an initial process of migration (diapedesis) of elements of blood corpuscles, such as neutrophil granulocytes, initially, and macrophages, subsequently. There is also increased plasma exudation in the interstitium. The exudate leads to a swelling of the area bordering the wound, the formation of which contributes to local acidosis [15,16,17,18,19,20,21,22]. The migration of leukocytes allows phagocytosis activity at the level of the lesion against pathogenic bacteria and damaged cells. In addition to phagocytosis, leukocytes are responsible for the production and secretion of numerous cytokines and growth factors essential for starting the subsequent phases of the healing process [15,16,17,18,19,20,21,22]. 

***Proliferative phase:*** The proliferative phase leads to the formation granulation tissue. During this stage, the fibroblasts play a central role, as they are responsible for the production of precursors of collagen, elastin, and other molecules fundamental to the constitution of the extracellular matrix, and are also implicated in the regulation of migration and proliferation of the cellular protagonists involved in the re-epithelialization process and neo-angiogenesis [15,16,17,18,19,20,21,22]. A fundamental role is also played by macrophages and MCs, which provide a continuous supply of growth factors necessary to stimulate angiogenesis. The mechanism of neo-angiogenesis is operated by the endothelial cells of the delimiting vessels at the lesion site, which, undergoing numerous mitotic cycles, give rise to new vessels capable of supplying trophic substances to the granulation tissue forming at the wound. At the epidermal level, on the other hand, the keratinocytes arranged at the edges of the lesion divide and begin to migrate towards the center of the injured site until the two edges rejoin, at which there is inhibition contact [15,16,17,18,19,20,21,22]. Once an abundant collagen matrix has been deposited in the wound, the fibroblasts stop producing collagen and the granulation tissue is replaced by a scar.

***Maturation phase:*** The remodeling of a wound can take up to 1 year. In humans, this phenomenon is characterized by two single processes, wound contraction and collagen restoration, where myofibroblasts allow contraction of the wound, with the formation of a scar both in children and adults [15,16,17,18,19,20,21,22]. During this process, the tensile strength increases, reaching approximately 80% that of unwounded skin, and is in relation to collagen crosslinking by lysyl oxidase [15,16,17,18,19,20,21,22].

## 4. PDT and Wound Healing

The mechanisms that lead to wound healing upon PDT treatment are not fully understood; however, one of the main reasons is represented by apoptosis, caused by damage to the cellular and mitochondrial membranes, enzymatic inactivation and arrest of cellular respiration processes, and the release of cytochrome C, leading to the activation of the caspase cascade. It has also been observed that PDT modulates the production of MMPs, cytokines, and growth factors by fibroblasts and keratinocytes, substances that can accelerate wound healing [26,27,28]. In particular, when the process of remodeling is required, MMPs are expressed and activated, and their contribution is related to collagen degradation and extracellular matrix remodeling [26,27,28].

Some authors have histologically assessed wounds treated with PDT: Mills et al. showed an improvement in matrix deposition in excision wounds [26], and Corsi et al., showed an increase in the thickness of the epidermis (demonstrated by the different location of the basal membrane), as well as that linked to the response of the inflammatory infiltrate [29,30]. An early onset of wound re-epithelialization after PDT has been described by studies in animal models, with the presence of young fibroblasts, fibrin, and granulation tissue [31]. 

Regarding the inflammatory process that develops [16,22], the occurrence of the following have been observed: the degranulation of MCs and neutrophil granulocytes [23,24,25,29,30], the formation of oxygen radicals, and the release of lysosomal enzymes and chemotactic agents. The release of antigens of dead cells, in the presence of inflammatory cytokines, determines the activation of skin DCs, which, after the presentation of these antigens to T lymphocytes in the district lymph nodes, stimulates a specific immune response [32].

Occurring simultaneously with the described cellular events, after PDT treatment, lipids are produced, as well as pro-inflammatory cytokines, such as IL-1β and IL-8, demonstrating that therapy has a significant effect on the immune system [26,27,28]. Moreover, since a balance between the synthesis and degradation of extracellular matrix is required, it is evident that PDT modulates the production of TGF-β [32], the isoforms of which are involved in the deposition of collagen fibers [26,27,28].

## 5. Chronic Wounds

Wounds that do not heal within 6/8 weeks are considered chronic [33,34,35,36,37]. Numerous factors prevent wound healing. Among local factors, it is necessary to acknowledge the presence of foreign bodies, tissue maceration, ischemia, infection, and tissue hypoxia. Among the systemic factors, advanced age, malnutrition, diabetes, and renal disease are, without doubt, factors of primary importance. In addition, reduction in the secretion of tissue growth factors, the decompensation between the proteolytic enzymes and their inhibitors, and the presence of senescent cells in the microenvironment seem to be particularly important in the pathogenesis of chronic wounds [33,34,35,36,37].

Chronic venous ulcers are associated with an extremely high psychosocial burden in terms of morbidity, loss of productivity, functional disability, and emotional distress, causing depression and social isolation. The difficulty, or even the impossibility, of treating these types of wounds leads to high costs, in terms of care, for the various communities [33,34,35,36,37].

In general, the processes involved in chronic wound healing are similar to those in acute wound healing, but their persistence leads to abundant granulation tissue and possibly fibrosis, scar contraction, and/or loss of function. Undoubtedly, MMPs, which can damage granulation tissue, are the most actively involved. During wound healing, cells in the injured area are induced by local mediators to secrete MMPs responsible of epithelization and proliferation. The dysregulation of MMPs is strongly associated with chronic wounds. In particular, increased expression of MMP-9 delays ulcer repair in diabetic patients via the activation of the ERK/AP1 signaling pathway [15,16,21,22].

Prolonged inflammation in chronic wounds [38] is mainly mediated by MCs, neutrophils, and DCs (including macrophages), which are attracted to the injured site, where they release pro-inflammatory and repair cytokines, and hydrolytic enzymes, which remove necrotic tissue, clean the wound, and prevent and resolve infection [16,21,24,32,38,39,40]. T cells take part in maintaining the pro-inflammatory profile of non-healing skin injuries [32]. Immune cells communicate with keratinocytes through the secretion of various signaling molecules. However, the contribution of these latter cells to the formation of a chronic wound is not fully understood [14].

## 6. PDT and Chronic Wounds

### 6.1. The Response of Cellular Infiltrate

Among the multiple properties of PDT, there is evidence of a strong cellular infiltrate response in the treated chronic wound (Figure 1C).

Moreover, in recent studies, it was found that, after PDT therapy in chronic wounds, there is a significant increase in certain inflammatory cells, such as TNF alfa+ MCs, T regs, plasmacytoid dendritic cells, MHCII positive dermal DCs [32], and macrophages [40], as well as an overall expression of TGF beta, which directly correlates with wound’s volume reduction [32]. TGF beta seems to exert activities in early phases of wound healing, where it possibly promotes an epithelial–mesenchymal transition, allowing the migration of keratinocytes from the borders towards the wound’s bed [41]. Finally, intercellular correlations between plasmacytoid dendritic cells and T reg have been found, confirming the fact that certain DC subsets are highly specialized in inducing regulatory T cell differentiation and, in some tissues, the local microenvironment plays a role in driving DCs towards a tolerogenic response [42,43].

Since TGF beta is also able to induce the differentiation of myofibroblasts as part of the other processes also seen in wound healing [44], in some studies [29,30], it has been reported that PDT-treated chronic wounds show an abundance of fibroblasts (Figure 1D) compared to controls and untreated wounds, providing evidence that one of the mechanisms of this therapy might be the alteration of inflammatory processes, presumably via the activation of the enzymatic systems produced by the target cells stimulated by PDT, leading to an eventual healing of the chronic wound. Since the secretion activity of fibroblasts (i.e., extracellular matrix) is stimulated by other factors present in the wound microenvironment, such as histamine by MCs [45,46], the close distances of these cells to fibroblasts (Figure 1D) and the expression of FGF in their granules, in PDT-treated wounds [30], confirms this hypothesis. Therefore, after PDT therapy, we can conclude that MCs may send signals for the recruitment and differentiation of fibroblasts, and these latter cells are involved in the healing process of chronic wounds.

Indeed, signals produced by MCs may, in turn, be delivered directly to other cellular types, such as dermal DCs (Figure 1E) [47,48], which are also directly involved in wound healing [49,50].

It has been established that, upon PDT therapy, MCs increase in number and undergo degranulation [22,29,30,32]. The origin of the increase in MC number is probably related to its response to the microenvironment, including the migration of other cells, the differentiation or influx of precursors, and their eventual transformation in MCs [24]. The time needed for the influx and differentiation of circulating precursors to MCs is not known exactly. Probably, MCs are not only recruited, but have to be activated to secrete in response to PDT treatment. The vessels of the papillary dermis appear to be an important site of cell infiltration and clustering upon therapy; consequently, it is presumable that endothelial cells, along with the recruitment of pericytes [51], can regulate the recruitment of MCs at this location [52].

### 6.2. Neuroimmunomodulation

In healing wounds, the activity of immune system is certainly modulated by the nervous system [53,54,55], and delayed wound healing is observed in animal models after surgical resection of cutaneous nerves [54,55]. Sensory neurons possess several means of detecting the presence of noxious or harmful stimuli: (1) cytokine receptors, such as IL-1β and TNFα, recognize the factors secreted by immune cells (e.g., IL-1β, TNFα, nerve growth factor), which activates MAP kinases and other signaling mechanisms to increase membrane excitability; (2) distress signal receptors, including TRP channels, P2X channels, and DAMPs, recognize exogenous signals from the environment (e.g., heat, acidity, chemicals) and signals endogenous hazards released during trauma or tissue injury (for example, ATP or uric acid) [56]. Studies have demonstrated that the stimulation of dorsal roots induces cutaneous vasodilation and enhancement of inflammatory processes [56], consisting of (a) chemotaxis and subsequent activation of neutrophils, macrophages, and lymphocytes at the site of injury; (b) degranulation of MCs; (c) an increase in blood flow, which also allows easier recruitment of inflammatory leukocytes; and (d) dendritic cell activation and subsequent T helper cell differentiation [32,38,57].

These observations clearly suggest that innervation and neuromediators play a pivotal physiological role in wound healing. Interactions between nerves and other cells involved in wound healing, such as MCs, are crucial in the healing process [24,56,58], and MCs are commonly observed in chronic wound samples [29,30,31]. An example of this functional relationship comes from a recent study [59], which investigated, in ALA-PDT-treated chronic wounds, MC interaction with neuronal cells containing neurotransmitters involved in wound healing processes, such as CGRP, NGF, NKA, NPY, SP, PGP 9.5, and VIP [53,54]. 

The results of this study [59] demonstrate that, in chronic wounds treated with ALA-PDT, there is an increase in neuronal populations containing mediators involved in wound healing, as well as that relating to the percentage of MCs containing NGF and VIP.

Since NGF and VIP stimulate MC degranulation [57,58], this last fact relates to an increase in the degranulation index of MCs after PDT treatment, as previously shown [29,30], and is probably related to nerve stimulation. Therefore, the effects of ALA-PDT therapy on chronic wounds, at least in this model, may probably be due to neuronal activation; therefore, nervous fibers can activate various cellular types during wound healing, including MCs [57,58]. 

The fact that MCs exhibit numerous interactions with nerve fibers [57,58], and that the VIP and NGF content in their granules increases [59] after treatment, is interesting. Keeping this in mind, at least in our model, it can be assumed that MC activity after therapy (i.e., their degranulation), probably due to a receptor [60], increases the release of NGF and VIP, which are able to interact with neurons and nerve fibers of the dermis, thus obtaining an improvement. The activation of nerve fibers could, in turn, be related to other phenomena, such as the increased secretion of extracellular matrix by fibroblasts, as has been observed previously [29,30], as well as increases in TGF beta levels [32] and the response of cellular infiltrates [29,30]. Of course, since these results derive from a single pilot study, further studies are needed to elucidate a direct correlation between clinic wound healing improvement and increased of local neuropeptides expression after ALA-PDT.

### 6.3. Future Perspective

Among neuronal mediators, particular attention should be directed towards nitric oxide, a neuromodulator involved in the control of vascular tone and blood pressure [61]. For example, iNOs is upregulated under stress conditions; in fact, in the presence of inflammatory cytokines and other agents (antigens of pathogens, apoptotic bodies, etc.), the expression of this enzyme increases, underlining its possible role in the inflammatory phase of wound healing, in which it could guarantee vasodilation and antibacterial activity. In our study [62], a strong response of iNOs following photodynamic therapy was reported, denoting how the latter actively participates in the improvement of the clinical condition of the wound. Experiments are underway in the laboratory to obtain further elucidation regarding this observation.

## 7. Current Limitations

All that has been presented in this review takes on great significance if we consider that photodynamic therapy is relatively young and, therefore, new indications for its use can be discovered in the future. As regards the effects of cellular mechanisms induced by photodynamic therapy on chronic wounds, the description of these events undoubtedly suffers from a certain immaturity, as the same chronic wounds still represent unresolved problems [63,64]. The cellular mechanisms still need to be tested before arriving at any official therapies. Certainly, the involvement of the nervous system and its interactions with the immune system must be looked at carefully and understood more fully, as they can be the key to the resolution of this type of wound if subjected to such therapy. 

## Figures and Tables

**Table 1:** Reagents used to stain inflammatory cells.

Substances	Target	References
HSP 47 (Antibody)	Fibroblasts	[11]
Avidin (Egg white protein linking biotin)	MCs	[12]
MHC class II (Antibody)	Dendritic cells	[13]

## Data Availability

Not applicable.

## Abbreviations

Acronym	Denomination
PDT	Photodynamic therapy
ROS	Reactive Oxygen Species
ALA	5-aminolevulinic acid
Matrix Metalloproteinases	MMPs
MCs	Mast Cells
Interleukin	IL
TGF	Transforming growth factor
ERK/AP1	ERK-associated changes of AP1
DCs	Dendritic cells
TNF	Tumor necrosis factor
BDCA	Blood dendritic cell antigen
HSP	Heat shock protein
FGF	Fibroblast growth factor
UEA	Ulex Europaeus Agglutinin
NGF	Nerve Growth Factor
MAP	Mitogen-activated protein
TRP	Transient receptor potential channel
P2X	ATP-gated P2X receptor cation channel family
DAMP	Damp-associated molecular pattern receptors
ATP	Adenosine triphosphate
CGRP	Calcitonin Gene Related Peptide
NKA	Neurokinin A
NPY	Neuropeptide Y
SP	Substance P
PGP 9.5	Protein Gene Product 9.5
VIP	Vasoactive intestinal peptide
iNOs	Inducible isoform of nitric oxide synthase
